# An annotated database of Arabidopsis mutants of acyl lipid metabolism

**DOI:** 10.1007/s00299-014-1710-8

**Published:** 2014-12-10

**Authors:** Kathleen McGlew, Vincent Shaw, Meng Zhang, Ryeo Jin Kim, Weili Yang, Basil Shorrosh, Mi Chung Suh, John Ohlrogge

**Affiliations:** 1 Department of Plant Biology, Michigan State University, East Lansing, MI 48824 USA; 2 College of Agronomy, Northwest A&F University, Yangling, Shaanxi 712100 People’s Republic of China; 3 Department of Bioenergy Science and Technology, Chonnam National University, Gwangju, 500-757 Republic of Korea; 4 3311 Laredo Lane, Fort Collins, CO 80526 USA

**Keywords:** Lipases, Regulatory mutants, ARALIP

## Abstract

*****Key message***:**

**We have constructed and annotated a web-based database of over 280 Arabidopsis genes that have characterized mutants associated with Arabidopsis acyl lipid metabolism.**

**Abstract:**

Mutants have played a fundamental role in gene discovery and in understanding the function of genes involved in plant acyl lipid metabolism. The first mutant in Arabidopsis lipid metabolism (*fad4*) was described in 1985. Since that time, characterization of mutants in more than 280 genes associated with acyl lipid metabolism has been reported. This review provides a brief background and history on identification of mutants in acyl lipid metabolism, an analysis of the distribution of mutants in different areas of acyl lipid metabolism and presents an annotated database (ARALIPmutantDB) of these mutants. The database provides information on the phenotypes of mutants, pathways and enzymes/proteins associated with the mutants, and allows rapid access via hyperlinks to summaries of information about each mutant and to literature that provides information on the lipid composition of the mutants. In addition, the database of mutants is integrated within the ARALIP plant acyl lipid metabolism website (http://aralip.plantbiology.msu.edu) so that information on mutants is displayed on and can be accessed from metabolic pathway maps. Mutants for at least 30 % of the genes in the database have multiple names, which have been compiled here to reduce ambiguities in searches for information. The database should also provide a tool for exploring the relationships between mutants in acyl lipid-related genes and their lipid phenotypes and point to opportunities for further research.

**Electronic supplementary material:**

The online version of this article (doi:10.1007/s00299-014-1710-8) contains supplementary material, which is available to authorized users.

## Introduction

New tools in science such as genome sequencing, RNASeq, microarrays, and metabolomics have provided enormous new data resources. Researchers are increasingly challenged to keep up with the avalanche of data and often feel overwhelmed. Fortunately, there are powerful search tools available to ‘mine’ many datasets, and there are excellent efforts to organize these data and to provide websites where specific data can be queried with sophisticated searches (Geneinvestigator, to name just one). However, the accumulation of new data almost always out-paces the ability to curate it, to integrate it with other databases, and to provide manual annotations by experts in the field.

**Fig. 1:**
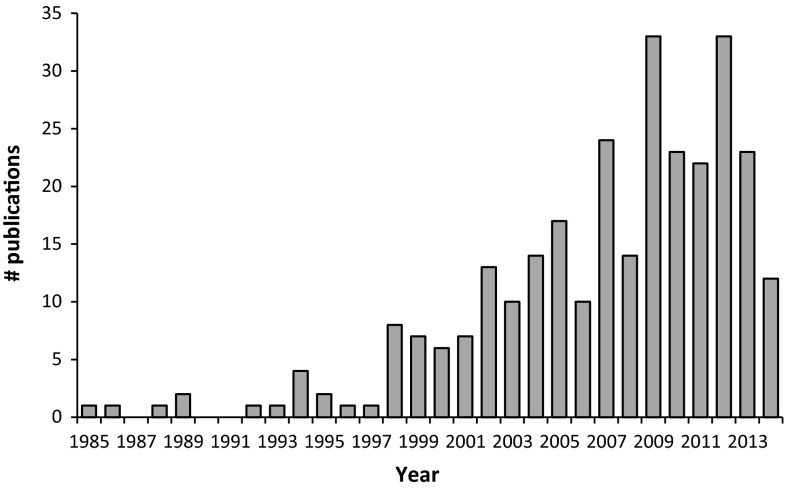
Arabidopsis lipid mutants reported in publications by year. Publications in which acyl lipid-related mutants were characterized in terms of function and phenotype were sorted by year. An increase in the number of new publications characterizing novel mutants may have peaked in 2012. The survey for 2014 included publications up to summer of that year

This review describes the results of an effort to organize, annotate, update, and curate key information about characterized mutants that impact Arabidopsis acyl lipid metabolism. The database/catalog (ARALIPmutantDB) has evolved out of the ARALIP website (http://aralip.plantbiology.msu.edu) and its underlying data and annotations. In addition, information from the excellent database published by Lloyd and Meinke (2012) has been incorporated. Approximately 20–30 studies on mutants of Arabidopsis acyl lipid metabolism have been published every year in recent years (Fig. 1). We have updated ARALIPmutantDB to include literature up to the summer of 2014. The database is available in Supplement Table 1, and updated versions can also be downloaded at http://aralip.plantbiology.msu.edu/downloads.

**Fig. 2:**
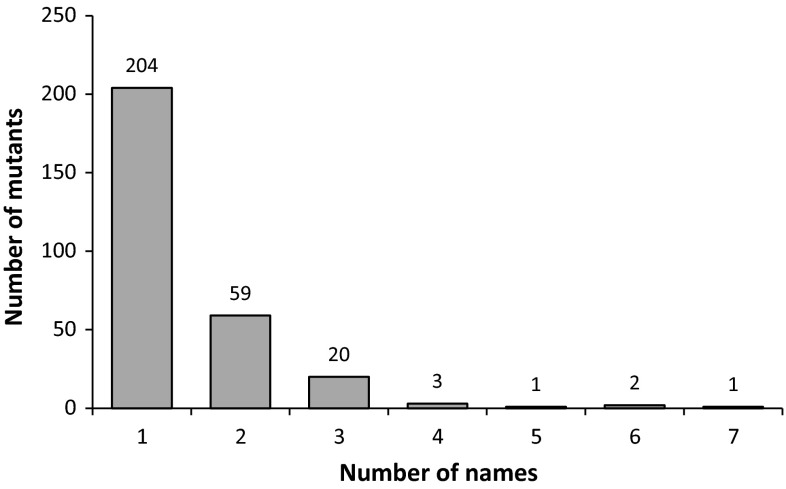
Prevalence of alternate names associated with Arabidopsis mutants of acyl lipid metabolism. 30 % of all mutants associated with a single gene in this survey have more than one name in the literature, with the highest number of names being seven. An alternative name was defined as a different name for mutants which have the same gene locus identification. Alternate names can arise for several reasons: from different alleles or because of changes in names (e.g., *act1 *= *ats1*) or because mutants in the same gene were given different names when discovered/characterized in different laboratories. Therefore, the same mutant could be described with several alternate names in different publications

The goals of this project have been to produce a catalog of mutants that is annotated by experts, that is linked to metabolic pathways, and that provides a resource to aid researchers in the field of plant lipid metabolism. This review provides a brief background on the identification of mutants in acyl lipid metabolism, and presents an overview of the database of these genes, and highlights some aspects of the distribution of mutants in different pathways and protein classes. The database provides information on the phenotypes of mutants, pathways, and enzymes/proteins associated with the mutants and allows rapid access via hyperlinks to summaries of information about each mutant and to references that provide information on the phenotypes and, in most cases, the lipid composition of the mutants. In addition, the database of mutants is integrated within the ARALIP plant acyl lipid metabolism website so that information about mutants is displayed on and can be accessed from over 15 metabolic pathway maps. At least 30 % of the genes in the database are associated with multiple mutants or mutants that have multiple names (Fig. 2). These have been compiled in the database to reduce confusion in searches for information. Overall, this database can provide a tool for exploring the relationships between mutants in genes and their lipid phenotypes. It is also hoped that knowledge of the lack of mutants for reactions, proteins, or pathways will help uncover opportunities for new insights.

## Forward and reverse genetic approaches for identification of mutants

The identification of mutants in Arabidopsis acyl lipid metabolism began with forward genetic screens pioneered by Chris Somerville. In 1985, Browse and Somerville reported in *Science* on the identification of a mutant (*fad4, At4g27030*) lacking *trans*-3 hexadecenoic acid (Browse et al. 1985). The mutant was identified by generating a population of EMS mutants and screening the fatty acid composition of leaves of approximately two thousand of these mutants by gas chromatography. It was initially very surprising to lipid biochemists that the *fad4* mutant had no visible phenotypes even though it lacked a fatty acid that had been conserved throughout more than 200 million years of plant evolution. After this landmark publication, using the same and similar approaches, the Somerville lab identified mutants that represented all major desaturases responsible for chloroplast and cytoplasmic glycerolipid unsaturation as well as acyl transferases and other enzymes (for review, see Wallis and Browse 2002).

Some mutants (e.g. *fad4*, *fad5*) impacted only the ‘prokaryotic’ lipids in the plastid (e.g., MGDG), whereas others changed the composition of extraplastidial lipids together with those lipids that were imported into the plastid from the ER (e.g., *fad2*, *fad3*). Other key mutants were also identified in these early studies. The *act1/ats1* mutant in the plastid acyl-ACP glycerol-3-phosphate acyltransferase was particularly illuminating because it resulted in a shift of flux from the prokaryotic to the eukaryotic pathway (Kunst et al. 1988). Thus, a major contribution of these mutants was that they provided crucial support and confirmation of the ‘two-pathway hypothesis’ of plant glycerolipid metabolism.

**Fig. 3:**
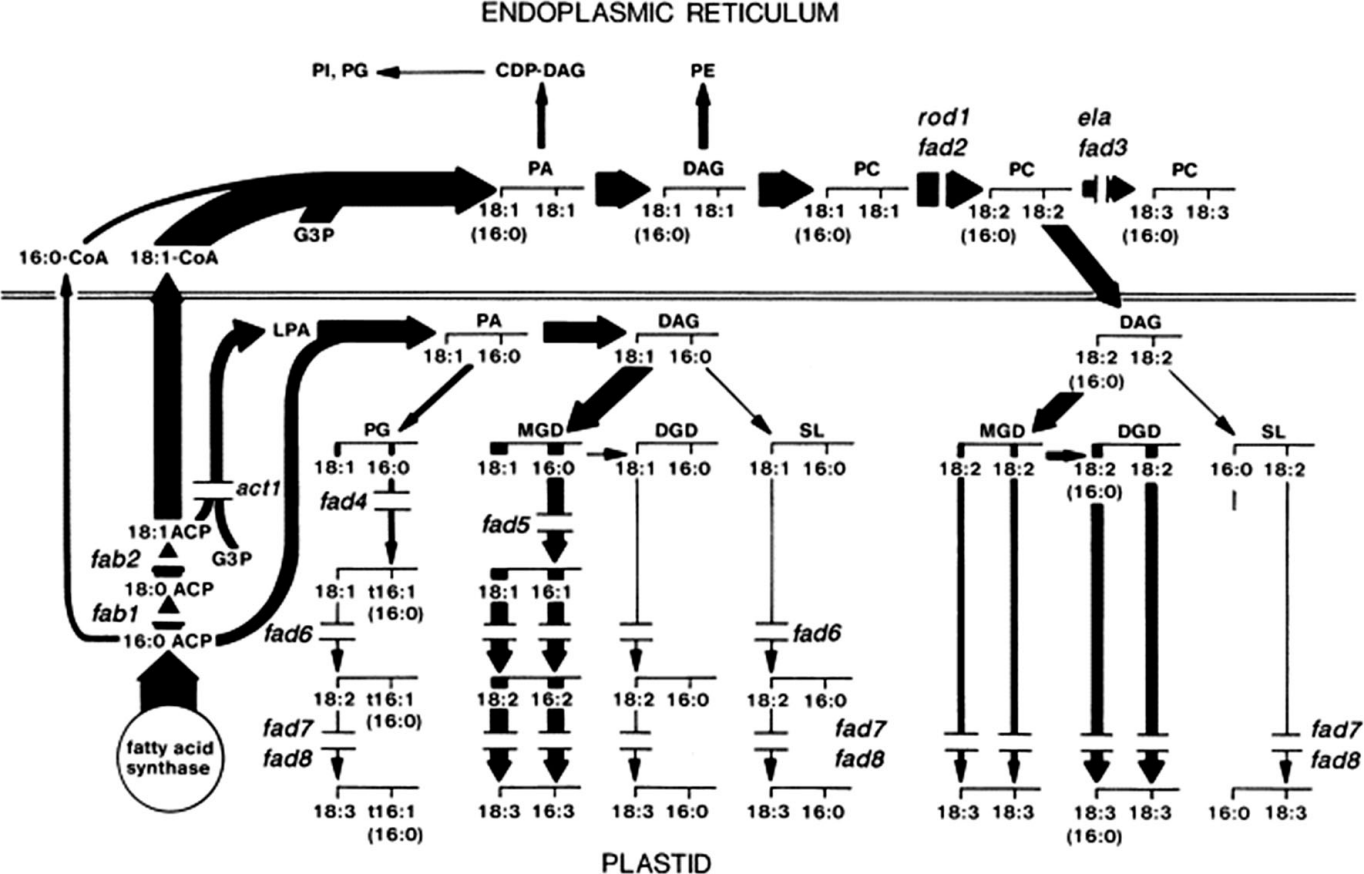
Mutants in Arabidopsis acyl lipid metabolism identified by forward genetics as of 1991. This figure is perhaps the most influential figure published in the field of plant acyl lipid metabolism. In addition to illustrating the mutants associated with acyl lipid metabolism, the width of arrows indicates flux through the prokaryotic and eukaryotic pathways of Arabidopsis leaves. Figure reproduced from Wallis and Browse (2002), and is based on an earlier version from Browse and Somerville (1991)

Progress as of 1991 with the forward genetics approach to Arabidopsis lipid metabolism was summarized in perhaps the most influential figure published in the field of plant acyl lipid metabolism (Fig. 3) (Browse and Somerville 1991). None of the genes that were responsible for the early mutant lipid phenotypes represented in Fig. 3 were known at the time of their publication. This changed with the historic first success in plant map-based cloning by Arondel et al. (1992), which resulted in the identification of the *FAD3* gene. Map-based cloning continues to be a major method for the identification of genes responsible for glycerolipid phenotypes. For example, it is interesting to note that the gene encoding FAD4 was not identified until more than 20 years after the mutant was first identified, and this success was achieved by map-based cloning in the lab of former Somerville student, Christoph Benning (Gao et al. 2009). In addition to the analysis of leaves, forward genetic screening was applied to Arabidopsis seeds, leading to major breakthroughs in the identification of genes crucial to the biosynthesis of seed oil. The *wrinkled1* mutant, with an 80 % reduction in seed oil, was identified by forward genetics and described by Focks and Benning (1998). The identification by map-based cloning of the *WRI1* gene as an AP2-type transcription factor was a landmark in understanding the control of seed oil biosynthesis (Cernac and Benning 2004). *Rod1* (*reduced oleate desaturation1*) was identified in a GC screen of seed fatty acids of EMS mutants (Lemieux et al. 1990). In 2009, map-based cloning of *rod1* led to the discovery of a new enzyme reaction, phosphatidylcholine: diacylglycerolcholine transferase (PDCT) (Lu et al. 2009).

**Fig. 4:**
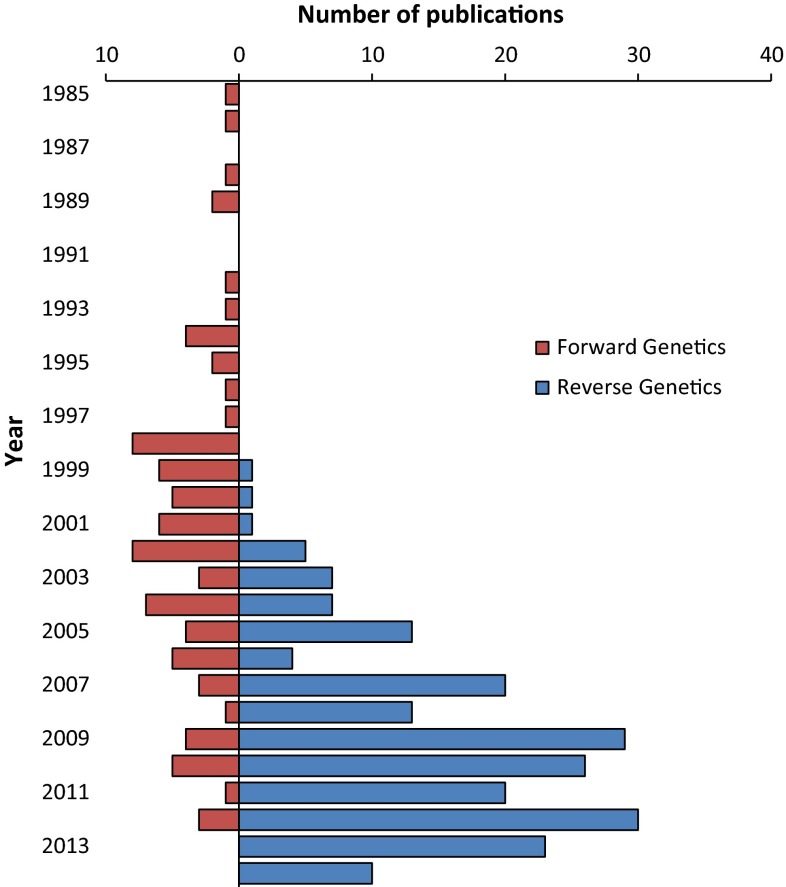
Changes in methods leading to identification for Arabidopsis mutants. Key publications which characterize acyl lipid-related mutants were sorted based on whether or not they were identified using forward or reverse genetic techniques. Designations of methods from publications before 2012 are based on Lloyd and Meinke (2012). More recent data is based on analysis of the literature from 2012 until July 2014. Note that many mutants were identified by a combination of forward and reverse genetics, which is not indicated here

Reverse genetics (identification of genes based on knowledge of DNA sequences) became possible at a large scale with the advent of high-throughput DNA sequencing and now is the most frequent method for establishing gene-mutant-phenotype relationships (Fig. 4). For Arabidopsis, a key advance was the sequencing of several thousand randomly selected cDNA clones [‘expressed sequence tags’ (ESTs)] reported in 1993 and 1994 (Höfte et al. 1993; Newman et al. 1994). This advance opened up the ability to ‘knock-down’ the expression of genes using RNA interference techniques and, equally important, to more readily identify candidate genes corresponding to mapped mutations. The number of ESTs expanded rapidly to over 100,000 by 2000, and these ESTs were estimated to represent approximately two-third of expressed genes of Arabidopsis. The publication of the Arabidopsis genome in 2000, in addition to greatly facilitating identification of genes by map-based cloning, encouraged the development of new reverse genetic tools, including the hugely valuable collection of T-DNA insertion mutants (Alonso et al. 2003), which later included sequence information at the site of insertion (O’Malley et al. 2007). Large-scale forward genetic screens of the T-DNA population, including many projects referred to as functional genomics, led to the discovery of plant acyl lipid metabolism genes (e.g., Ajjawi et al. 2010, 2011). Over the past 15 years, reverse genetics has become increasingly facile and has accounted for over 80 % of gene-mutant characterizations since 2005 (Fig. 4).

The identification of the gene encoding diacylglycerol acyltransferase (*DGAT1*) was a major contribution to the understanding of seed oil metabolism, and illustrates an example of forward genetics being aided by DNA sequence information from other organisms and from cDNA clones. The *tag1*mutant (*as11*) was obtained by EMS mutagenesis and GC screening of seed fatty acid composition. Further characterization of *as11* indicated a reduction in diacylglycerol acyltransferase activity (Katavic et al. 1995). In 1999, two labs reported the sequence of the gene responsible for loss of DGAT activity in *tag1*-*1* (*as11*) (Zou et al. 1999) and in *tag1*-*2* (*abx45*), an independently identified low oil mutant from a T-DNA insertion population (Routaboul et al. 1999).

Although gene-mutant connections are now most frequently made via reverse genetics (Fig. 4), forward genetic approaches have continued to provide crucial new breakthroughs, particularly in the discovery of genes where candidate sequences could not be predicted based on the previous work. Thus, forward genetics has led to many of the most novel discoveries. Examples include discovery of four genes required for transport of lipids from the ER to the plastid (Benning 2009), and the discovery that *cer7* is a subunit of a RNA processing and degrading exosome (Hooker et al. 2007).

This survey undoubtedly has missed many mutants in acyl lipid metabolism that have been studied. In some cases, authors of ARALIP or of this review simply were not aware of the published work. In many more cases, mutants have displayed little or no phenotype and this information is either not published, or information on the mutants is presented in ways that it is not easily found. For example, Zhang et al. (2009a) describe a number of acyltransferase mutants that have no seed oil phenotype, but these negative results only appear in one sentence. The database is therefore biased (as is the literature) toward mutants with clear biochemical phenotypes. There are also datasets from ‘functional genomic’ screens in which large populations of mutants have been analyzed for phenotypes, including lipids. These include, for example, a survey of leaf fatty acid composition of more than 5000 T-DNA insertion mutants (Ajjawi et al. 2010). Because most results from such surveys need to be further validated, we have not included these results unless they are described in full publications. To improve the database, we request our colleagues in the plant lipid community to submit corrections and additions to the authors or via the comment form at: http://aralip.plantbiology.msu.edu/forms.

## Results and discussion

### Development of the database

To create a central database that includes almost all acyl lipid-associated mutants and related information, we consolidated several sources. We started with data from the Arabidopsis Acyl-Lipid Metabolism website (ARALIP: http://aralip.plantbiology.msu.edu), an annotated database of lipid-related genes known or suspected to participate in Arabidopsis acyl lipid metabolism. Specific annotations in this database were provided by 27 authors of the Acyl Lipid Metabolism chapter of The Arabidopsis Book (Li-Beisson et al. 2013). 152 gene loci that were associated with a characterized mutant were identified from the ARALIP database.

We further identified mutants by comparing the ARALIP database to a dataset of Arabidopsis genes with a loss-of-function mutant phenotype that is provided in a key publication by Lloyd and Meinke (2012). By comparing the two lists, we identified 70 additional mutants that were curated by Lloyd and Meinke but that were not included in ARALIP. The Lloyd and Meinke list included details on the phenotypic classifications and descriptions of mutants that were not already in ARALIP, and some of this information has been incorporated into ARALIPmutantDB. Comparing these sets also indicated that the original ARALIP database includes approximately 60 mutants not in the Lloyd and Meinke set.

The ARALIP website and the Lloyd and Meinke database were based on the data up to 2012 and 2011, respectively, with publication dates of 2013 and 2012. To provide more recent information, we searched the literature to identify additional mutants described between January 2012 and summer of 2014. The major search tool was the ISI Web of Science, using the search words “Arabidopsis,” “lipid,” and “mutant.” Full text searches with Google Scholar retrieved too many unrelated references and Textpresso had not been updated to include references from 2014 at the time of the search; these were therefore less effective than ISI. We also consulted colleagues in the field for relevant publications from 2012 onward. In this way, we added an additional 68 mutants identified from publication in 2012–2014. Over 280 genes with characterized mutants are now included in ARALIPmutantDB compared to the original list of 152 in ARALIP. Thus, mutants have been characterized for approximately 30 % of the more than 900 genes in the 2013 version of the ARALIP database. This contrasts with the fact that, as of November 2014, approximately 90 % of all Arabidopsis loci are associated with T-DNA insertions (The Arabidopsis Information Resource). We searched a TAIR listing of loci without T-DNA insertions and found that only 20, or approximately 2 % of ARALIP genes, do not have an associated T-DNA insert (Table S2). Thus, there is a ‘reservoir’ of T-DNA insertion mutants that may provide information on the functions of many more ARALIP genes. Of the 20 ARALIP genes without known T-DNA insertions, 8 have associated mutants that were identified without the benefit of T-DNA insertions. Intriguingly, 6 of the remaining 12 loci are annotated as lipid transfer protein (LTP) and 4 as oleosins. These might represent attractive targets for further investigation.

We identified key references for each mutant in this database by using both ARALIP and Lloyd and Meinke’s (2012) list of references as a starting point. We then searched the literature for references that characterized the mutant phenotype. The earliest reference listed for each mutant is useful because it often provides the best information on lipid composition; information that is often difficult to find with available search tools, particularly when much of the data are presented in figures or tables in older publications. The earlier references also provide the best tool for tracking new publications based on citations. Other related references are also linked to the gene locus in the ARALIP databases.

It is very common for mutants or different mutant alleles of the same gene to have multiple names, and this occurs for 30 % of genes in ARALIPmutantDB, with the highest number of names being seven (Fig. 2). An alternative name was defined as a different name for mutants which have the same gene locus identification. Alternate names can arise for several reasons: from different alleles or because of changes in names (e.g., *act1* was updated to *ats1*) or because mutants in the same gene were given different names when discovered/characterized in different laboratories. The use of different names can cause confusion in discussions, in the literature, and can lead to ambiguities in searches for information on the mutants which results in researchers missing key information. This is particularly true for new researchers entering the field. Thus, by compiling the names we hope to improve understanding of the large amount of literature on plant acyl lipid mutants.

**Fig. 5:**
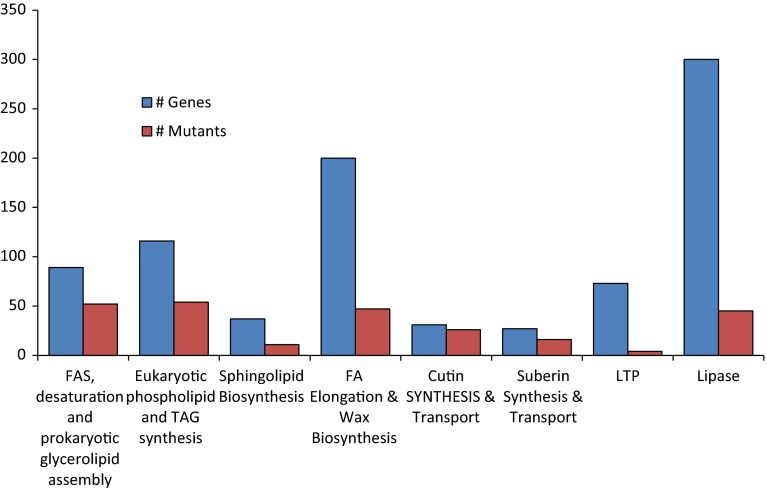
Mutants characterized by pathway. 293 lipid-related mutants in ARALIPmutantDB were sorted based on their involvement in different ARALIP pathways or protein type. The LTP family is not included in wax biosynthesis but instead is shown separately. Eukaryotic phospholipid and TAG synthesis has the highest number of mutants at 54, which account for 47 % of the total records of genes in that pathway. In contrast, a very small proportion of genes annotated as LTP or lipase have characterized mutants. Note that some genes are associated with multiple pathways

The ARALIP website and database are organized with genes that are classified as belonging to different metabolic pathways. Some genes fit into more than one category. Figure 5 displays the proportion of genes in a few selected pathways that have characterized mutants. Eukaryotic phospholipid and TAG synthesis have the highest number of mutants at 50, which account for 45 % of the total records of genes assigned to these pathways. Other categories are striking in the low proportion of mutants. For example, there are 74 genes annotated as lipid transfer proteins (LTP) but mutants have been characterized for only 10 of these. In addition, over 300 genes in Arabidopsis include the term “lipase” in their annotation. However, we could find literature describing mutants for only 50 of these genes (discussed below).In the following sections we present a brief analysis of two areas of plant lipid research where the proportion of mutants identified compared to the number of genes is particularly low, and thus may offer opportunities for new discoveries. Our hope is that by highlighting these areas, other workers in the field will be attracted to the opportunities they present for new discoveries. In these sections, the genes which are associated with mutants are indicated by italicized locus IDs.

### Arabidopsis putative lipases: why are there so many?

A large number of Arabidopsis genes that are annotated with the term “lipase” were originally not included in the ARALIP database because their function and catalyzed reaction is usually poorly characterized, if at all. However, to contribute to the general need for greater understanding of these genes, we expanded ARALIPmutantDB to include genes that include “lipase” in TAIR annotations [selected based on the lists presented in Troncoso-Ponce et al. (2013)]. Supplement Table 3 provides a summary of the distribution of characterized mutants within the different classes of lipases.

A major enigma in the study of plant lipid metabolism is why there may be so many genes involved in lipid breakdown or turnover. It is striking that there are more putative lipase genes than genes for the central anabolic pathways for fatty acid synthesis and glycerolipid assembly. However, experimental evidence confirming the *in planta* roles of these genes as lipases is available for only approximately 30–40 genes (Li-Beisson et al. 2013). The further exploration of these putative lipases may be a path toward new insights. Indeed major recent discoveries have included the role of a GDSL lipase (Yeats et al. 2012, 2014) and of an *α/β* hydrolase (Jakobson et al. 2014) in cutin synthesis. It is important to note that the proposed role of these genes is to catalyze acyltransferase reactions, rather than lipase reactions.

Lipases which have been annotated to date are grouped here into phospholipases (PL), phosphatidic acid phosphatase (PAP), triacylglycerol lipases (TAGL), monoacylglycerol lipases (MAGL), GDSL lipases, and proteins harboring an α/β hydrolase motif. A large number of these annotations are based only on sequence homology to enzymer from other organizms and have not been confirmed to be correct for the Arabidopsis genes.

Phospholipids are not only major structural components of membranes, but also a reservoir for lipid-signaling molecules. Phospholipases (PLs) are enzymes that catalyze the hydrolysis of phospholipids either at the acyl ester bond, to release fatty acids, or at the polar head group. There are four major classes, termed A, B, C and D, which are distinguished by the type of reaction which they catalyze. PLA is divided into PLA1 and PLA2 that cleave the *sn*-1 and *sn*-2 acyl chains, respectively. PLB hydrolyzes both *sn*-1 and *sn*-2 acyl chains and is also known as a lysophospholipase. PLC hydrolyzes the O–P bond adjacent to the glycerol, releasing diacylglycerol and a phosphate-containing head group. PLD hydrolyzes the O–P bond adjacent to the head group, releasing PA and a head group.

PLA is divided into two groups, which are secreted and patatin-related PLAs. The first putative PLA1 targeted into the plastid was identified from characterization of a *defective in anther dehiscence 1*(*dad1, At2g44810*) mutant, which is defective in the biosynthesis of jasmonic acid (JA) in flower buds (Hyun et al. 2008; Ishiguro et al. 2001). A homolog of *DAD1*, *DONGLE  *(DGL, At1g05800), which is expressed in vegetative tissues was identified to be involved in the early phase of wound-inducible JA biosynthesis, whereas DAD1 functions in the late phase of wound-inducible JA production. DAD1 has a preferential substrate specificity for DGDG, suggesting that the major source of JA is the acyl chains derived from DGDG  (Hyun et al. 2008). *Shoot gravitropism 2* (*SGR2*, *At1g31480*) encodes a PLA1 involved in shoot gravitropism, and it has been suggested that the *SGR2* might be important in the formation of membrane structure (Kato et al. 2002). The PLA2 subfamily is known to have four genes, *PLA2α, β*, *γ*, and *δ*. *PLA2β* (*At1g53940*) as like *SGR2* is also involved in cell elongation and shoot gravitropism (Lee et al. 2003). The role of *PLA2*β*, γ* (*At4g29460*), and *δ* in the formation of membrane structure was discovered during pollen development and germination by the suppression of PLA2 (Kim et al. 2011). The suppressor of *AvrBsT*-*elicited resistance1* (*SOBER1*, *At4g22300*) regulates PA levels generated in Arabidopsis in response to biotic stress (Kirik and Mudgett 2009). In addition, the patatin-related PLAs (pPLAs) are grouped into three subfamilies; pPLAI, pPLAII (*α*, *β*, *γ*, *δ*, and *ɛ*), and pPLAIII (*α*, *β*, *γ*, and *δ*). pPLAs preferentially hydrolyze membrane glycerolipids such as monogalactosyl monoacylglyceride (MGDG) and phosphatidyl-glycerol (PG). pPLAs and the released fatty acids have been implicated in plant growth and stress responses. pPLAI was implicated in maintaining the levels of basal JA that plays a positive role in defense responses (Yang et al. 2007). *pPLAIIα*-deficient plants were observed to have higher levels of JA, methyl-JA, and the oxylipin-biosynthetic intermediates than in wild type, indicating that *pPLAIIα* (*At2g26560*) might function in the removal of oxidatively modified fatty acids in membranes for membrane repair or remodeling (Yang et al. 2012). *pPLAIIβ* (*AtPLAIVB/PLP5*, *At4g37060*) is involved in root elongation under phosphate deficiency, and pPLAII*γ* and *δ* were implicated in auxin responses (Rietz et al. 2010). Among four *pplaIIIα* (*At5g43590*)*, β* (*At2g39220*)*, γ* (*At3g54950*), and *δ* (*At3g63200*) knock-out mutants, only *pplaIIIδ*-*KO* seeds exhibited significant increase in seed oil contents with 20- and 22-carbon fatty acids, suggesting that *pplaIIIδ,* which is able to hydrolyze PC, may play a role in fatty acyl flux from plastid to the ER and/or PC fatty acyl remodeling for TAG synthesis (Li et al. 2013).

Possible functions of PLC have been identified in response to abiotic stresses. Non-specific phospholipase C5 (*NPC5*, *At3g03540*) is reported to be involved in galactolipid accumulation by providing DAG intermediates in leaves under phosphate limitation condition (Gaude et al. 2008). The *npc4* mutant (*At3g03530*) shows decreased DAG levels, decreased ABA sensitivity in seed germination, root elongation and stomatal movement, and decreased resistance to drought and salt stresses, suggesting that NPC4 and DAGs promote stomatal opening under well-watered conditions and PA promotes plant growth under water-deficit conditions (Peters et al. 2010). Phosphoinositide (PI)-specific *PLC9* (*At2g40116*) is involved in thermotolerance of Arabidopsis, suggesting that the released IP3 may regulate intracellular Ca^2+^ concentration, which could ultimately induce genes encoding heat shock proteins (Zheng et al. 2012).

Among 12 PLDs including PLD*α* (3), *β* (2), *γ* (3), *δ*, *ɛ*, and *ξ* (2), *in planta* roles of 8 PLDs have been characterized. PLD and its product PA are implicated in many cellular processes including plant growth and development, plant responses to abiotic and biotic stresses, hormone responses, and vesicle trafficking. PA produced by *PLDα1* (*At3g15730*) binds to NADPH oxidase, which stimulates ROS production in guard cells, showing that PA is a signaling molecule for ABA-mediated stomatal closure (Zhang et al. 2009b). *PLDα1* (*At3g15730*) and *PLDδ* (*At4g35790*) cooperatively function in ABA-induced stomatal closure and seed germination (Uraji et al. 2012; Distefano et al. 2012). *PLDα3* (At5g25370) is involved in plant growth and development, particularly flowering, under water-deficit conditions (Hong et al. 2008). The *pldβ 1* mutant (*At2g42010*) showed an increased resistance to the bacterial infection (*Pseudomonas syringae* pv. DC3000), but was more susceptible to fungal infection (*Botrytis cinerea*) compared with wild type, suggesting that *PLDβ1* and associated lipid changes are involved in the SA-dependent and JA/ethylene-dependent plant defense (Zhao et al. 2013). PA produced by *PLDδ* (*At4g35792*) is involved in cell wall-based defense signaling in non-host resistance against powdery mildew fungi via hydrogen peroxide production (Pinosa et al. 2013). *PLDɛ* (*At1g55180*) was found to enhance plant growth under high salinity and water deficiency, suggesting that *PLDɛ* and PA may play a positive role in nutrients such as nitrogen signaling (Hong et al. 2009). *PLDξ* (*At3g05630*) and PA enhance vesicle trafficking and positively regulate auxin responses by regulating the activities of RCN1 (a protein phosphatase 2A regulatory subunit) and PID1 (a Ser/Thr protein kinase), which are a potential target protein of PA and a regulator of polar auxin transporter, respectively (Li and Xue 2007).

PA phosphatase (PAP) catalyzes the dephosphorylation of PA to diacylglycerol (DAG). Arabidopsis contains four genes encoding membrane-bound PAP (also called lipid phosphate phosphatase, LPP), two genes encoding soluble and cytosolic PAP (also called PA phosphohydrolase1 and 2), and three genes encoding plastidic PAP. Among 4 LPPs, disruption of *LPP2* (*At1g15080*) caused hypersensitivity to ABA and significant PA accumulation during seed germination, suggesting that PA is involved in ABA signaling (Katagiri et al. 2005). The *pah1pha2*-*1* mutant exhibited a significant increase in phospholipid content, up-regulation of genes encoding enzymes involved in phospholipid synthesis, and alteration in ER morphology, suggesting that PAH/2 may play a role in repressing phospholipid synthesis at the ER or may modulate phospholipid synthesis through changes in the level of PA or DAG (Eastmond et al. 2010). Based on sequence homology searches of *LPP* genes from *Cyanobacterium synechosystis* sp. PCC6803, 3 putative plastidic Arabidopsis *LPP* genes {*LPPγ* (*At5g03080*), *LPPɛ1* (*At3g50920*), and *LPPɛ2* (*At5g66450*)} were identified. The *lppɛ1 lppɛ2* mutant showed no significant changes in lipid composition, whereas loss of *LPPγ* may cause a lethal effect on plant viability (Nakamura et al. 2007).

TAG hydrolysis is required for the mobilization of storage reserves during seed and pollen germination, and may be involved in membrane lipid remodeling during leaf senescence, and in plant defense against bacterial pathogen or insect attack. Arabidopsis *PAD4* (*At3g52430*) and *MLP1* (*At5g14180*), which display sequence similarity to TAG lipase, have been identified to be involved in plant defense (Jirage et al. 1999; Louis et al. 2010). Overexpression of *SAG101* (*At5g14930*), a leaf senescence-associated gene causes premature leaf senescence, whereas its antisense lines showed delayed leaf senescence. The SAG101 fusion proteins showed an acyl hydrolase activity for triolein (He and Gan 2002). *AtLip1* (*At2g15230*) with the best homology to human gastric lipase and *SDP1* (*Sugar*-*dependent 1, At5g04040*) encoding a patatin-domain TAG lipase were reported to be involved in storage oil breakdown during seed germination (Karim et al. 2005; Eastmond 2006). In addition, monoacylglycerol lipase (MAGL) catalyzes the hydrolysis of MAG to fatty acid and glycerol, the last step of TAG breakdown. Among 16 Arabidopsis genes annotated as MAGL, only 1 gene, lysoPL2/*AtMGAT* (Arabidopsis monoacylglycerol acyltransferase, *At1g52760*) has been characterized by mutant analysis. *lysoPL2* promotes the degradation of lysoPC in response to cadmium-induced oxidative stress (Gao et al. 2010) and *AtMGAT* was reported to have both monoacylglycerol acyltransferase and acyl hydrolase activities (Vijayaraj et al. 2012). Expression patterns, subcellular localizations, and enzyme activities of 16 Arabidopsis MAGLs have been recently characterized (Kim et al. 2012).

GDSL lipase containing a GDSL motif is a >100 member gene superfamily of possible lipolytic enzymes that collectively appear to display very broad substrate specificity. *GDSL lipase 1* (*GLIP1*), *GLIP2*, *Tomato Cutin Deficient1* (*CD1*), and *Arabidopsis cutin synthase1* (*AtCUS1*) genes belong to this very large family. In the analysis of Arabidopsis secretome in response to salicylic acid, *GLIP1* (*At5g40990*) was reported to be involved in plant immunity against fungal and necrotropic pathogens, suggesting that *GLIP1* may play a role in ethylene-associated systemic immunity (Oh et al. 2005; Kim et al. 2014). *GLIP2* (*At1g53940*) was also involved in resistance to necrotropic bacteria, *Erwinia carotovora* via negative regulation of auxin signaling (Lee et al. 2009). After a tomato GDSL lipase, *Cutin Deficient 1* (*CD1*) was identified to be an acyltransferase in cutin synthesis (Yeats et al. 2012), putative Arabidopsis homologues (also called cutin synthase, CUS) of *CD1*/*SlCUS1* (*Solanum lycopersicum* cutin synthase 1) were searched and *AtCUS1*/*LTL1* demonstrated cutin synthase activity in vitro (Yeats et al. 2014).

Other lipases that contain a *α*/*β*-hydrolase motif include EDS1, BDG, CGI-58, and PES1 and 2. *Enhanced disease susceptibility1* (*EDS1*, *At3g48090*) is involved in a disease-resistance process conditioned by TIR-NB-LRR type R gene that encodes a leucine-rich repeat protein (Falk et al. 1999). Bodyguard (*BDG*, At1g64670) is associated with cuticle development and morphogenesis (Jakobson et al. 2014; Kurdyukov et al. 2006). A mutant defective in the Arabidopsis *CGI*-*58* homologue (also called *ABHD5,* or *α*/*β*-*hydrolase*-*5*, *At4g24160*) caused accumulation of lipid droplet in leaves, suggesting that *CGI*-*58* may play a role in neutral lipid homeostasis in plants (James et al. 2010). *Phytyl* *Ester Synthase1* and *2* (*PES 1* and *2*, *At1g54570* and *At3g26840*) containing both *α*/*β*-hydrolase and acyltransferase motifs function in the formation of phytyl esters in chloroplasts (Lippold et al. 2012).

Several of the examples presented above make it clear that the original annotations of genes as “lipase”, PLA, etc. need to be revised/updated as new information on their actual in vivo enzymatic activity becomes available.

### Mutants of transcription factors and other regulators of acyl lipid metabolism

Over the past several decades, considerable information has become available on regulation of lipid metabolism by transcription factors (TFs) and other regulators, and is well summarized in recent reviews (Lee and Suh 2013; Li-Beisson et al. 2013; Marchive et al. 2014; Borisjuk et al. 2014). However, considering there are over 1800 total TFs in Arabidopsis, it is perhaps surprising that such a small number have been shown to regulate acyl lipid metabolism. Some regulators have been identified in other plants but not in Arabidopsis, such as *SHOOTMERISTEMLESS* (*STM*), *TT16*, *bZIP123* (Deng et al. 2012; Song et al. 2013). Possible reasons for the low number are that the functions of regulators may be redundant, such that the phenotypes in single mutants are masked by complementation by other genes, such as two negative regulators of oil, the High-level expression of *Sugar*-*Inducible gene 2* (*HSI2*, *At2g30470*) and *HSI2*-*Like1* (*HSL1*, *At4g32010*) (Tsukagoshi et al. 2007), or that mutations in regulation of critical primary metabolic pathways are lethal.

With more than 35 % oil in its seeds, Arabidopsis provides one of the best model plants for seed oil biosynthesis. Several transcription regulators involved in TAG accumulation have been identified. Forward genetic methods were used in early studies to find mutants with altered oil contents. Focks and Benning (1998) reported the isolation of a mutant named *wrinkled1* (*At3g54320*), which has 80 % reduction in oil content and in flux of carbon through glycolysis and fatty acid synthesis. The identification of the *WRI1* gene represents a landmark advance in studies of plant lipid metabolism (Cernac and Benning 2004) and its role is now by far the best understood of any TF in acyl lipid metabolism (Marchive et al. 2014). *WRI1* binds to an upstream AW-box region of genes involved in fatty acid synthesis, such as *pyruvate kinase* (*Pl*-*PKb1*) and acetyl-CoA carboxylase (BCCP2) (Maeo et al. 2009). It is now known that *WRI1* directly binds to a large number of target genes in the glycolytic and fatty acid biosynthesis pathways (Marchive et al. 2014). WRI3 (At1g16060**)** and WRI4 (At1g79700), two other WRI1-like proteins can complement the *wri1* mutant, are expressed in flower and stem, and may regulate cutin synthesis indirectly through control of fatty acid synthesis (To et al. 2012).

LEC1 (At1g21970), LEC2 (At1g28300), FUSCA3 (FUS3,At3g26790) and ABI3 (At3g24650) are master regulators in embryo development and deficiencies of oil were found in their mutants (Finkelstein and Somerville 1990; Keith et al. 1994; Lotan et al. 1998; Stone et al. 2001). PKL (At2g25170), a chromatin-remodeling factor, represses LEC1 expression in vegetative tissues and oil accumulates in the primary roots of *pkl* mutant (Ogas et al. 1997, 1999). ABI4 (At2g40220), another member of APETALA2 TFs, plays a role in the degradation of storage oil and the expression of DGAT1 is down-regulated in its mutant (Penfield et al. 2006; Yang et al. 2011). GLABRA2 (GL2, At1g79840), a homeodomain (HD) TF, acts as a negative regulator of seed oil (Shen et al. 2006). Several other negative regulators of TAG accumulation have been recognized by reverse genetic strategies in recent studies. TT2 and TT8, regulators of proanthocyanidin and flavonoid synthesis in testa, also affect oil contents and fatty acid compositions in seeds. FUS3, CAC2 (At5g35360), KASII (At1g74960), FAD2 (At3g12120) and FAE1 (At4g34520) are up-regulated in *tt2* (*At5g35550*) and *tt8* (*At4g09820*) mutants (Chen et al. 2012, 2014). ASIL (At1g54060) belongs to the trihelix family of DNA-binding TFs and depresses the expressions of genes related to embryo-specific lipids as well as embryo development (Gao et al. 2011). Additionally, several TFs have been identified as regulators of fatty acid compositions of seed oil. The bZIP67 (At3g44460) targets to FAD3 (At2g29980) and 18:3 levels are reduced in seed oil of *bzip67* mutant (Mendes et al. 2013), while total fatty acid compositions are changed differentially in the seeds of *crc* (*At1g69180*) and *ap1* (*At1g69120*) mutants (Han et al. 2012). PII (GLB1, At4g01900), directly controlled by *WRI1*, finely adjusts fatty acid composition in seeds (Baud et al. 2010).

The plant cuticle is the protective layer coating the aerial surface of higher plants, which mainly consists of cutin and cuticular wax. Several transcription factors have been confirmed to control the synthesis of cuticular lipids. SHINE1/WAX INDUCER1 (SHN1/WIN1, At1g15360), an AP2-domain protein, was first identified to regulate cutin synthesis by coordinate induction or by direct interaction with genes known to be involved in cutin deposition, such as *long chain acyl*-*CoA synthetase2* (*LACS2*, *At1g49430*) (Aharoni et al. 2004; Broun et al. 2004; Kannangara et al. 2007). SHN2 (At5g11190) and SHN3 (At5g25390), two other members of the SHINE clade of AP2 TFs, also regulate cutin- and suberin-associated genes, such as *CYP86A cytochrome P450* *s* and fatty acyl-CoA reductases (Shi et al. 2011). Additional AP2 TFs affecting cuticle deposition include GLABRA1 and GLABRA3 (Xia et al. 2010). TTG1, a WD40 repeat protein also has been shown to affect cuticle deposition (Xia et al. 2010). The Curly Flag Leaf1 (CFL1, At2g33510), a WW domain TF, negatively regulates cuticle development by modulating the function of HDG1 (At3g61150) (Wu et al. 2011). As noted above, WRI3 and WRI4, two other WRI1-like proteins expressed in non-seed tissues, are likely indirect regulators of cutin synthesis through fatty acid synthesis (To et al. 2012). DEWAX (At5g61590), an AP2/ERF-type TF, negatively regulates the expressions of genes related to surface lipid synthesis. More wax accumulates on the surface of stems and leaves of *dewax* mutant (Go et al. 2014). After MYB30 (At3g28910) and MYB41 (At4g28110) were identified as regulators of surface lipids (Cominelli et al. 2008; Raffaele et al. 2008; Kosma et al. 2014), more members of MYB family have been confirmed in recent studies. MYB106 (At3g01140) and MYB16 (At5g15310), cooperate with SHN1 to regulate epicuticular wax crystals and cutin nanoridges (Oshima et al. 2013). MYB96 (At5g62470) activates a group of wax biosynthesis genes, such as very long chain fatty acid-condensing enzymes (Seo et al. 2011). MYB41 positively regulates the steps necessary for aliphatic suberin synthesis and deposition of cell wall-associated suberin-like lamellae (Kosma et al. 2014). Cer7 (At3g60500) is a core subunit of the RNA processing/degrading exosome and influences wax biosynthesis by controlling the expression of CER3/WAX2/YRE (Hooker et al. 2007). A screen for suppressors of the *cer7* mutant revealed regulatory roles for RNA-DEPENDENT RNA POLYMERASE1 (RDR1) and SUPPRESSOR OF GENE SILENCING3 (SGS3) in CER7-mediatied RNA silencing of CER3 (Lam et al. 2012). Similarly, *SERRATE (At2g27100)* encodes a protein of RNA–processing multi-protein complexes thought to mediate “RNA signaling in the cuticle integrity pathway” (Voisin et al. 2009). *HUB1* (At2g44950) and *HUB2* (At1g55250) encode two orthologous RING E3 ligases and wax and cutin compositions are altered in *hub* mutants (Menard et al. 2014). Similarly, *CER9 (At4g34100)* “encodes an E3 ubiquitin ligase homologous to yeast Doa10 (previously shown to target endoplasmic reticulum proteins for proteasomal degradation)” thought to serve as a negative regulator of cuticle synthesis and stress responses (Lu et al. 2012). More general regulators of embryo epidermal differentiation have been implicated in regulating cuticle development including ZHOUPI (At1g49770), ALE1 (At1g62340), GASSHO1 (At4g20140), GASSHO2 (At5g44700) and ACR4 (At3g59420). (Tsuwamoto et al. 2008; Tanaka et al. 2002; Watanabe et al. 2004; Cao et al. 2005; Xing et al. 2013).

Although a few regulators have been described to impact membrane lipids, many of these may do so indirectly. Bax inhibitor-1 (At5g47120), an endoplasmic reticulum protein, regulates sphingolipid synthesis by interacting with four related enzymes (Nagano et al. 2014). GOLDEN2-LIKE1 (GLK1, At2g20570) and GLK2 (At5g44190), regulators of chlorophyll synthesis, also regulate the expressions of galactolipid-synthesis genes, particularly DGD1 under continuous light (Kobayashi et al. 2014). ARF7 (At5g20730) and ARF19 (At1g19220) are auxin-response TFs and the accumulation of DGDG and SQDG is suppressed in *arf7arf19* mutant during phosphate starvation (Narise et al. 2010).

In addition to *WRI1* and *bZIP67* mentioned above, a small number of studies have identified direct targets of TFs by DNA binding assays. LEC2 directly binds with RY motifs in the 5′ flanking regions of some LEC2 induced genes (Braybrook et al. 2006). ChIP-tiling array results demonstrate that FUS3 directly targets to many embryogenesis related TFs and microRNAs (Wang and Perry 2013). ChIP assay suggests that TT8 binds to the promoter regions of LEC1, LEC2, FUS3 and Cytidinediphophate diacylglycerol synthase2 (CDS2) (Chen et al. 2014). MYB96 (At5g62470), confirmed by both in vivo and in vitro assays, binds to the promoters of 3-ketoacyl-CoA synthase 1 (KCS1, At1g01120), KCS2 (At1g04220), KCS6 (At1g68530), KCR1 (At1g67730), and lipid transfer protein3 (LTP3, At5g59320) and positively adjusts their expression (Seo et al. 2011; Guo et al. 2013). ABI4 binds the coupling element1 (CE1) element [CACC (G)] in the promoter of its target genes in maize and Arabidopsis (Niu et al. 2002; Yang et al. 2011). LACS2 was reported as a direct target of WIN1 by immunoprecipitation assay (Kannangara et al. 2007). Clearly, much more is needed to clarify the mechanism of the other regulators mentioned above.

## Summary/conclusions

A few comments and observations derived from ARALIPmutantDB.

1. There are some large gene families where a surprisingly small number of mutants have been identified. The lipid transfer protein (LTP) gene family is one intriguing example. There are 73 genes in this family, but fewer than 10 % of these genes have characterized mutants. The GDSL lipase family, with over 100 members has only 2 genes in ARALIPmutantDB. Of course, it is important to remember that many genes annotated as lipases may not in fact act on lipids or catalyze hydrolase reactions.
2. Desaturases were some of the first enzymes where mutants were characterized and now all major desaturase genes in central pathways of plant glycerolipid desaturation likely have been identified and characterized with mutants. However, several genes in the ‘ADS’ or FAD5-like class have uncertain functions and may be attractive candidates for further focus.
3. Although WRI1, WRI3 and WRI4 are clearly the master regulators of fatty acid synthesis in seed and non-seed tissues, there are a number of remaining questions. Triple *wri1 wri3 wri4* mutants still accumulate oil at 20 % of WT levels, raising the question of whether there are other transcription factors in play for oil synthesis of developing seeds. These three transcription factors are expressed at low levels in tissues such as leaves; although young, rapidly expanding leaves synthesize fatty acids at rates approaching those of seeds. Thus, a related question is: what controls production of fatty acids/lipids in leaves, where WRI is not highly expressed?
4. Although WRI1 clearly regulates the expression of a number of genes in glycolysis and fatty acid synthesis and this determines oil content of seeds, very little is known about control of expression of genes later in the TAG assembly pathway. DGAT1, PDAT1 and other acyltransferases have patterns of expression during seed development that are strikingly different than those of the fatty acid biosynthesis pathway (e.g., Troncoso-Ponce et al. 2011). What TF or other factors are responsible for controlling their expression?
5. How close are we to having identified most genes that have a lipid phenotype in single mutants? The number of publications on new mutants in Arabidopsis acyl lipid metabolism may have peaked (Fig. 1). This might reflect the fact that most genes with readily detectable mutant phenotypes have been characterized or that gene redundancies ‘conceal’ the gene function in mutants.

### **Author contribution statement**

JO and MCS conceived and designed the project. KM and VS collected and analyzed information and created the figures and database. WY collected information, added annotations, and edited the manuscript. BS designed and created the web version of the database. KM, MZ, RJK, MCS, and JO wrote the manuscript. All authors read and approved the manuscript.

## Electronic supplementary material

Below is the link to the electronic supplementary material.

Supplementary material 1 (XLSX 138 kb)
